# Prevalence and Impact of Concomitant Atrial Fibrillation in Patients Undergoing Percutaneous Coronary Intervention for Acute Myocardial Infarction

**DOI:** 10.3390/jcm13082318

**Published:** 2024-04-17

**Authors:** Iqra Shakeel, Harish Sharma, James Hodson, Hamna Iqbal, Rashna Tashfeen, Peter F. Ludman, Richard P. Steeds, Jonathan N. Townend, Sagar N. Doshi, M. Adnan Nadir

**Affiliations:** 1 College of Medical and Dental Sciences, Institute of Cardiovascular Sciences, University of Birmingham, Birmingham B15 2TT, UKharish.sharma@nhs.net (H.S.); hamnaiqbal42@gmail.com (H.I.); rashna.t@hotmail.co.uk (R.T.); rick.steeds@uhb.nhs.uk (R.P.S.); john.townend@uhb.nhs.uk (J.N.T.); sagar.doshi@uhb.nhs.uk (S.N.D.); 2 Department of Cardiology, University Hospitals Birmingham, Birmingham B15 2TH, UK; 3 Research Development and Innovation, University Hospitals Birmingham NHS Foundation Trust, Birmingham B15 2GW, UK

**Keywords:** Atrial fibrillation, myocardial infarction

## Abstract

**Background:** Concomitant atrial fibrillation (AF) is associated with an adverse prognosis in patients with acute myocardial infarction (MI). However, it remains unclear whether this is due to a causal effect of AF or whether AF acts as a surrogate marker for comorbidities in this population. Furthermore, there are limited data on whether coronary artery disease distribution impacts the risk of developing AF. **Methods:** Consecutive patients admitted with acute MI and treated using percutaneous coronary intervention (PCI) at a single centre were retrospectively identified. Associations between AF and major adverse cardiac and cerebrovascular events (MACCEs) over a median of five years of follow-up were assessed using Cox regression, with adjustment for confounding factors performed using both multivariable modelling and a propensity-score-matched analysis. **Results:** AF was identified in N = 65/1000 (6.5%) of cases; these patients were significantly older (mean: 73 vs. 65 years, *p* < 0.001), with lower creatinine clearance (*p* < 0.001), and were more likely to have a history of cerebrovascular disease (*p* = 0.011) than those without AF. In addition, patients with AF had a greater propensity for left main stem (*p* = 0.001) or left circumflex artery (*p* = 0.004) involvement. Long-term MACCE rates were significantly higher in the AF group than in the non-AF group (50.8% vs. 34.2% at five years), yielding an unadjusted hazard ratio (HR) of 1.86 (95% CI: 1.32–2.64, *p* < 0.001). However, after adjustment for confounding factors, AF was no longer independently associated with MACCEs, either on multivariable (adjusted HR: 1.25, 95% CI: 0.81–1.92, *p* = 0.319) or propensity-score-matched (HR: 1.04, 95% CI: 0.59–1.82, *p* = 0.886) analyses. **Conclusions:** AF is observed in 6.5% of patients admitted with acute MI, and those with AF are more likely to have significant diseases involving left main or circumflex arteries. Although unadjusted MACCE rates were significantly higher in patients with AF, this effect was not found to remain significant after adjustment for comorbidities. As such, this study provided no evidence to suggest that AF is independently associated with MACCEs.

## 1. Introduction 

Atrial fibrillation (AF) is the most frequent sustained cardiac arrythmia [1], and its prevalence is increasing worldwide as life expectancy increases [2,3,4]. Atrial fibrillation has an association with coronary artery disease (CAD) and myocardial infarction (MI) due to shared risk factors and an inflammatory aetiology [5,6]. The reported prevalence of concomitant AF in patients following acute MI varies widely, with reported rates ranging between 6 and 21% [7,8]. Previous studies have identified AF as an independent risk factor both for MI [7,8,9,10] and for mortality after MI [11,12,13,14,15,16,17]. However, it is unclear whether this is due to a causal effect of AF or whether the observed effect is a result of AF acting as a surrogate marker for other comorbidities that influence the outcome. There is also a paucity of data on long-term outcomes beyond 12 months. Furthermore, there are no data on the pattern of CAD in patients with AF compared to those in sinus rhythm presenting with acute MI. 

Therefore, the aims of this large cohort study were as follows: (i) to determine the prevalence of AF in acute MI patients treated with percutaneous coronary intervention (PCI) at the time of admission, (ii) to determine whether AF is an independent prognostic factor with respect to the long-term risk of major adverse events, and (iii) to compare the distribution of CAD in patients with and without AF. 

## 2. Methods

### 2.1. Data Collection

Consecutive patients admitted to the Queen Elizabeth Hospital Birmingham (QEHB) with MI and treated with PCI between January 2016 and November 2017 (inclusive) were retrospectively identified from a prospectively maintained and validated database. Data relating to demographics, medical history, presentation, treatment, and discharge medications were obtained from the electronic health record (EHR). Details of the MI included the type of MI (ST-Elevation Myocardial Infarction [STEMI] or Non-ST-elevation Myocardial Infarction [NSTEMI]), as well as both the high-sensitivity troponin T (Roche Cobas E170) levels recorded at the time of the index admission and the peak level during the admission. The coronary angiography findings were then used to determine the patterns and distribution of CAD across the left anterior descending artery (LAD; proximal or distal), right coronary artery (RCA), and left circumflex artery (LCx). The pattern of CAD was then summarised based on the number of these vessels with severe disease, defined as ≥75% luminal stenosis. Involvement of the left main stem (LMS) was also assessed, with severe disease defined as a ≥50% luminal stenosis. Measurements of left ventricular ejection fraction (LVEF) were extracted from the report of the echocardiogram performed closest to the date of the PCI for each patient; LVEF ≥ 50% was classified as normal, with 40–49%, 30–39%, and <30% classified as mild, moderate, and severe levels of LVEF impairment, respectively. 

The primary factor of interest was AF, which was diagnosed from the 12-lead surface electrocardiogram (ECG) performed during the index admission, based on established diagnostic criteria [18]. In cases where ECGs were unobtainable, the discharge letters and subsequent outpatient clinic letters were interrogated to identify any evidence that AF had been diagnosed at the index admission. For patients with AF, the EHR was further interrogated to identify whether this had been pre-existing at the time of their admission and whether it represented chronic or paroxysmal AF. 

The primary outcome of interest was the development of major adverse cardiac and cerebrovascular events (MACCEs), which was a composite outcome comprising the following: death, MI, cerebrovascular accident (CVA), or major gastrointestinal (GI) bleeding. To identify where this outcome occurred, all readmissions to QEHB occurring after the date of PCI were identified from the EHR. From these, the International Classification of Diseases (ICD10) codes recorded for each readmission were interrogated to identify any that related to the MACCE events; the ICD10 codes used to identify each of these outcomes are reported in Supplementary Table S1. In addition, the EHR was used to extract the dates of death in patients that died during the follow-up period. 

### 2.2. Statistical Methods

Comparisons between the AF and non-AF groups were performed using Mann–Whitney U tests for continuous or ordinal variables, with Fisher’s exact tests being used for nominal variables. For time-to-event outcomes, follow-up commenced at the date of PCI, with patients being censored on the date of data extraction (4 April 2022). The primary outcome was MACCE, which was a composite of death and readmission for any of the previously described adverse events (whichever came first). However, the individual components of MACCEs were also analysed separately using a death-censored approach. Analyses were initially performed using Kaplan–Meier curves, which were used to estimate cumulative event rates. Associations between other factors and the primary outcome of MACCEs were then assessed using univariable Cox regression models. For continuous factors, the goodness of fit was assessed graphically, with factors being divided into categories and treated as nominal where poor fit was detected. A multivariable Cox regression model was then produced, with AF entered at the first step and a backwards stepwise approach (removal at *p* > 0.1) used to select other factors for inclusion in a parsimonious model. 

Adjustment for confounding factors was also performed using a propensity score matching approach. A propensity score was first produced using a multivariable binary logistic regression model, with AF acting as the dependent variable. For categorical factors with missing data for more than one AF patient, an “unknown” category was added to prevent excessive exclusions. Given the relatively small number of patients with AF, variable selection used a backwards stepwise approach to minimise the risk of overfitting. However, a more lenient threshold for removal of *p* > 0.2 was used in an attempt to minimise the risk of underfitting. The factors selected for inclusion by the stepwise procedure were then entered into a new model to prevent exclusions of patients with missing data for factors not included in the propensity score model. The predicted probabilities from the resulting model were then logit-transformed and used to match patients from the AF group 1:1 to patients in the non-AF group, using a calliper of ±0.2 times the standard deviation of the logit. Cohort characteristics were then compared between the matched groups using Wilcoxon’s signed-rank test for continuous or ordinal variables and either McNemar or McNemar–Bowker tests for nominal variables with two or more than two categories, respectively. Rates of MACCE were compared between groups using a univariable Cox regression model, which was stratified by the pair number, to account for the matching of AF and non-AF patients. 

All analyses were performed using IBM SPSS 24 (IBM Corp. Armonk, NY, USA), with *p* < 0.05 deemed to be indicative of statistical significance throughout. Continuous variables are reported as means ± standard deviations where approximately normally distributed or as medians (interquartile range; IQR) otherwise. Cases with missing data were excluded from the analysis of the affected variable for univariable analysis, with multivariable analyses using a complete-cases approach.

## 3. Results

### 3.1. Cohort Characteristics

The study cohort comprised N = 1000 patients with MI, of whom N = 65 (6.5%) presented with concomitant AF. In those with AF, this had been newly diagnosed at the index admission in 48% (31/65) of cases, with 72% (47/65) having chronic AF. Patients with AF were significantly older than those in the non-AF group (mean: 73 vs. 65 years, *p* < 0.001) and had lower creatinine clearance (mean: 67 vs. 85 mL/min, *p* < 0.001) and a higher burden of comorbidities, including diabetes mellitus (*p* = 0.028) and hypertension (*p* = 0.009), as well as higher rates of previous CVA (*p* = 0.011), MI (*p* = 0.003), and PCI (*p* = 0.029). In addition, AF was associated with significantly higher rates of LVEF impairment (*p* = 0.008, Table 1). 

There were no significant differences between the AF and non-AF groups with respect to revascularisation time (between symptom onset and PCI; median: 36 vs. 24 h, *p* = 0.179), rates of out-of-hospital cardiac arrest (*p* = 0.334), type of MI (NSTEMI or STEMI; *p* = 0.365), or peak high-sensitivity troponin levels (median: 800 vs. 512 ng/L, *p* = 0.155). However, mitral regurgitation (MR) was significantly more common in those with AF (55.4% vs. 27.0%, *p* < 0.001). 

Patients with AF were significantly more likely to undergo coronary artery bypass grafting (CABG; 9.2% vs. 3.3%, *p* = 0.028). Discharge medications also differed significantly between groups (Table 2), with patients with AF being significantly more likely to receive clopidogrel, non-vitamin-K-antagonist oral anticoagulants, warfarin, aldosterone receptor antagonists, and diuretics and significantly less likely to receive aspirin, ticagrelor, or an angiotensin-converting enzyme inhibitor. 

### 3.2. Patient Outcomes

**Figure 1:**
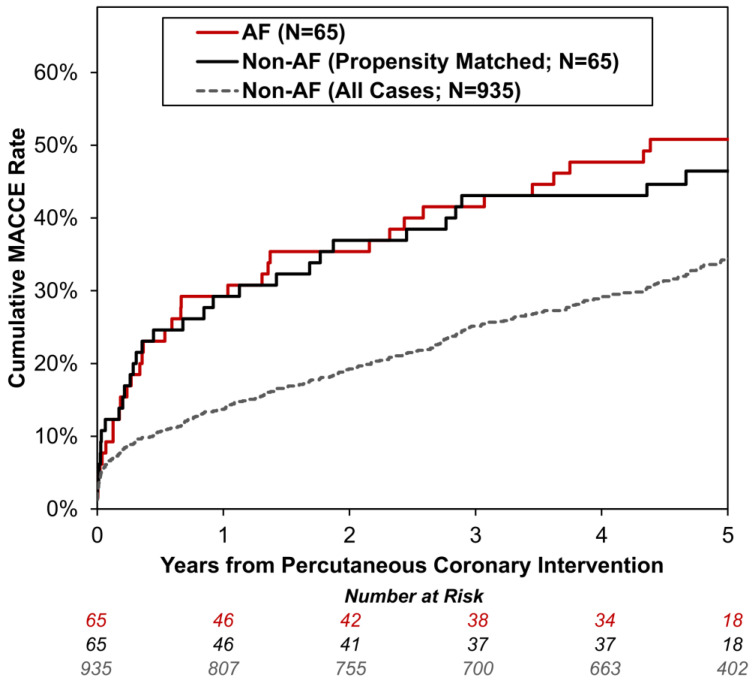
**Kaplan–Meier curves of major adverse cardiac and cerebrovascular events sorted by concomitant atrial fibrillation.** For the non-AF group, separate curves are plotted for the group as a whole (N = 935), as well as for the subgroup of patients that were propensity-score-matched to the AF group (N = 65; Supplementary Table S2). The x-axis is truncated at five years of follow-up. AF: Concomitant Atrial Fibrillation, and MACCE: Major Adverse Cardiac and Cerebrovascular Event (defined as death or readmission due to myocardial infarction, cerebrovascular accident, or major gastrointestinal bleed).

Patients were followed up for a median of 60 months (IQR: 53–67), during which time there were N = 258 deaths, and N = 168 patients had at least one readmission for MI, CVA, or a major GI bleed. MACCE rates were significantly higher in AF patients compared to the non-AF group, with an unadjusted hazard ratio (HR) of 1.86 (95% CI: 1.32–2.64, *p* < 0.001), and the AF and non-AF groups had estimated rates at five years of 50.8% and 34.2%, respectively (Figure 1). Unadjusted univariable analysis for the individual components of MACCEs found patients with AF to have both significantly reduced survival (HR: 2.25, 95% CI: 1.54–3.29, *p* < 0.001) and significantly higher (death-censored) composite MI/CVA/major GI bleed readmission rates (HR: 1.86, 95% CI: 1.11–3.11, *p* = 0.018) than the non-AF group. Analyses of death-censored readmission rates for MI, CVA, and major GI bleeds separately returned similar HRs although none of these analyses reached statistical significance due to the low event rates (Table 3).

### 3.3. Predictors of Major Adverse Cardiac and Cerebrovascular Events

A multivariable model was then produced to assess whether AF was independently associated with MACCEs after adjusting for the effects of confounding factors (Table 4). This found increasing age (*p* < 0.001), lower creatinine clearance (*p* = 0.011), insulin-dependent diabetes mellitus (*p* < 0.001), hypertension (*p* < 0.001), previous CVA (*p* < 0.001) or MI (*p* = 0.004), increasing levels of LVEF impairment (*p* = 0.009) or MR severity (*p* = 0.042), and triple-vessel CAD (*p* = 0.010) to be significant independent predictors of MACCEs. After adjusting for the effects of these factors, no significant association between AF and MACCEs was observed (adjusted HR: 1.25, 95% CI: 0.81–1.92, *p* = 0.319).

**Figure 2:**
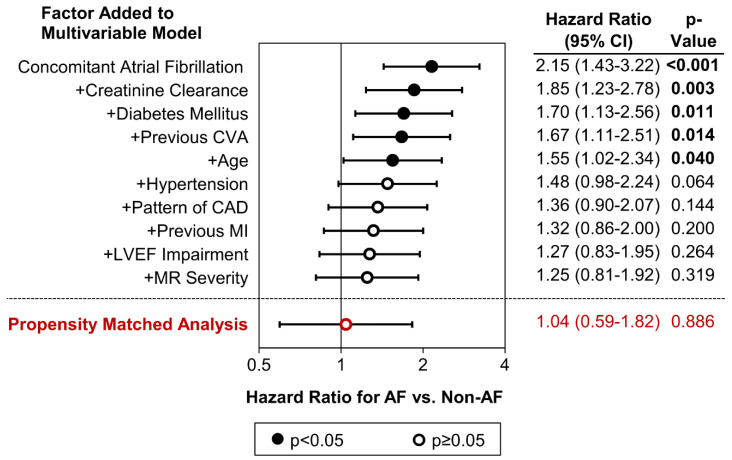
**Effect of concomitant atrial fibrillation on major adverse cardiac and cerebrovascular events after adjustment for confounding factors.** The first section of the plot illustrates the effect of adjustment for confounding factors using a multivariable analysis. The first point represents the hazard ratio and 95% CI for AF vs. non-AF in a Cox regression model with MACCE as the only dependent variable. This presentation differs from that reported for the univariable analysis in Table 3 since all analyses in the plot use a complete-cases approach to missing data; hence, all hazard ratios are based on N = 772 cases (N = 263 events) as per the multivariable analysis in Table 4. Confounding factors were then added iteratively to this model using a forwards stepwise approach. The subsequent points on the plot represent the adjusted hazard ratios for AF vs. non-AF patients from each of these models, which include the stated factor, and the cumulative set of factors from the previous models. The second section of the plot illustrates the hazard ratio and 95% CI for AF vs. non-AF patients from a stratified Cox regression model of the propensity-core-matched cohort (N = 65 pairs of patients; Supplementary Table S2). AF: Concomitant Atrial Fibrillation, 95% CI: 95% Confidence Interval, CAD: Coronary Artery Disease, CVA: Cerebrovascular Accident, LVEF: Left Ventricular Ejection Fraction, MACCE: Major Adverse Cardiac and Cerebrovascular Event (defined as death or readmission due to myocardial infarction, cerebrovascular accident, or major gastrointestinal bleed), MI: Myocardial Infarction, and MR: Mitral Regurgitation.

In an attempt to explain the non-significance of AF in this model, the multivariable analysis was repeated using an iterative approach. AF was initially included in the model in isolation, before other factors were added progressively using a forwards stepwise approach, to assess how accounting for each additional factor influenced the adjusted effect of AF (Figure 2). This did not identify any individual factor as being influential in negating the observed effect of AF on MACCEs; instead, the adjusted effect of AF became progressively smaller as more factors were added to the model. This implies that the significance of AF in univariable analysis was largely a result of the confounding effects of baseline differences between the AF vs. non-AF groups rather than a causal effect of AF on MACCEs.

Adjustment for the baseline differences between the AF and non-AF groups was also performed using a propensity-score-matched approach. The propensity score considered all factors from Table 1 for inclusion and was used to match each AF patient 1:1 with a non-AF patient. Comparisons between these matched groups identified no significant differences in demographics or presentation, indicating that the matching had successfully negated the key baseline differences between the groups (Supplementary Table S2). Specifically, the AF vs. matched non-AF groups were similar with respect to age (mean: 73 vs. 71 years, *p* = 0.191) and creatinine clearance (mean: 67 vs. 66 mL/min, *p* = 0.966) and in the rates of diabetes mellitus (45% vs. 37%, *p* = 0.543), previous CVA (12% vs. 17%, *p* = 0.607), MR (55% vs. 63%, *p* = 0.435), and LMS involvement (18% vs. 22%, *p* = 0.804). No significant association between AF and MACCEs was observed for the matched groups, with a HR of 1.04 (95% CI: 0.59–1.82, *p* = 0.886) and cumulative rates of MACCEs at five years of 51% vs. 46% for the AF and matched non-AF groups, respectively (Figure 1).

### 3.4. Associations of AF with Coronary Artery Disease Distribution Pattern

**Figure 3:**
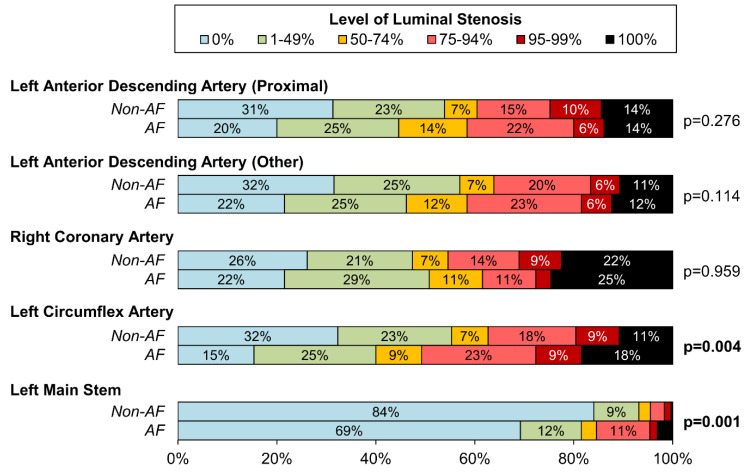
**Level of luminal stenosis sorted by vessel and concomitant atrial fibrillation.** Comparisons between the AF and non-AF groups were performed using Mann–Whitney U tests, and bold *p*-values were significant at *p* < 0.05. Unlabelled bars comprise <5% of cases. AF: Concomitant Atrial Fibrillation.

Patients with AF had a significantly greater level of luminal stenosis in the LMS (≥50% stenosis in 18.5% vs. 6.8%, *p* = 0.001) and the LCx (≥75% stenosis in 50.8% vs. 37.3%; *p* = 0.004; Figure 3). There were no significant differences between the AF and non-AF groups in the level of luminal stenosis in the LAD (proximal: *p* = 0.276; non-proximal: *p* = 0.114) or RCA (*p* = 0.959). The patterns of CAD across the LAD, RCA, and LCx were also similar in the two groups (*p* = 0.322, Table 1), with severe disease observed in at least two vessels for 43.1% vs. 36.0% of the AF vs. non-AF groups.

## 4. Discussion

This study found that AF is observed in 6.5% of type 1 acute MI patients revascularised through PCI and is associated with a higher prevalence of severe disease in the LMS and LCx on invasive coronary angiography. The presence of AF was not found to be a significant independent risk factor for MACCEs but instead appears to be a surrogate marker of other risk factors including older age, worse renal function, insulin-dependent diabetes mellitus, hypertension, prior CVA or MI, LVEF impairment, and MR. The study design uniquely focussed on acute MI patients managed by revascularisation through PCI (which accounted for the majority of patients), thus excluding those with type II MI or MINOCA or those managed medically. It also addresses the paucity of data relating to coronary artery distribution and AF prevalence, as well as long-term outcomes (median of five years of follow-up) for both mortality and the composite outcome of MACCEs.

The reported prevalence of AF in this study was similar to those of some previous studies [11,16] but was less than those reported in other studies [19]. This may be due to geographic variations or methodological differences in AF identification. This study also contradicted an earlier study that found that the presence of AF was not associated with the localisation of CAD [20]. Previous studies have found that the involvement of the LMS and atrial branches increases the likelihood of developing AF post-MI [21] and, as the left atrial branch is supplied by the LCx, we theorise that the co-existence of AF and severe CAD within the LMS and LCx may be due to left atrial hypoperfusion promoting remodelling and fibrosis. Further studies including the use of cardiovascular magnetic resonance are required to investigate this relationship further.

Finally, this study provided some data on the prognostic role of AF, which has been the subject of conflicting data in the post-MI population. We found that AF is a surrogate marker of disease in patients with MI rather than an independent risk factor. This was in contrast to previous studies [11,12,13,14,15,16,17], and our study demonstrated this over a longer period of follow-up and in patients with predominantly preserved LVEF. A recent study from Japan explored the differences between pre-existing and newly-diagnosed AF and found that although AF was not an independent risk factor for MI, it was independently associated with long-term mortality and stroke. It concluded that the adverse impacts of AF were similar between pre-existing and new-onset AF groups, except for the risk of stroke, which was higher with newly diagnosed AF [22].

## 5. Limitations

The authors acknowledge the limitations of this retrospective study. The selection of patients from our catheterisation laboratory database allowed a large sample size of acute MI patients to be identified. However, this resulted in only acute MI patients managed by revascularisation through PCI being included; hence, the results cannot be generalised to patients managed medically or surgically. Furthermore, despite the large sample size, the number of patients with AF was relatively small, which would have resulted in only moderate statistical power. As such, if AF had had a small but genuine independent association with MACCEs, then this may not have been detectable in the analysis, leading to a false-negative error. In addition, although several methods were used to determine the presence of AF (inpatient 12-lead ECGs, discharge letters, and outpatient clinic letters), it is possible that some episodes of paroxysmal AF were overlooked. There were also significant differences in demographics between the AF and non-AF groups, confounding the unadjusted analysis of prognosis. Adjusted analyses were performed to negate the impact of confounding using two different approaches (multivariable modelling and propensity score matching), which returned consistent results. However, these models could not adjust for unmeasured or intangible factors and, as such, the results may have been subject to residual confounding. Finally, the number of patients with detected AF receiving oral anticoagulation on discharge was lower than expected (approximately 50%). This may have been due to patients not meeting the threshold for anticoagulation or the presence of contraindications.

## 6. Conclusions

In conclusion, AF is associated with a significantly increased risk of MACCEs. However, our analysis suggests that this association is largely not causal but instead reflects the confounding effects of the increased burden of comorbidities associated with AF. For the first time here, we showed that this holds true for long-term outcomes. Since AF appears to be a surrogate marker of other risk factors, including hypertension, diabetes mellitus, and more severe CAD, patients with AF require tailored aggressive treatment for risk factor modification. Ambulatory cardiac monitoring in acute MI patients is currently not widely performed. A further prospective study with the routine ambulatory monitoring of all MI patients would help to validate the findings of this study and shape future practice.

## Figures and Tables

**Table 1:** Cohort characteristics organized by concomitant atrial fibrillation.

		Concomitant Atrial Fibrillation		
	N	No	Yes	*p*-Value
Patient Demographics				
Age (Years)	1000	65 ± 13	73 ± 11	**<0.001**
Gender (% Male)	1000	671 (71.8%)	49 (75.4%)	0.571
Smoking Status	963			0.155
Non-		332 (36.7%)	24 (40.7%)	
Ex-		300 (33.2%)	24 (40.7%)	
Current		272 (30.1%)	11 (18.6%)	
Creatinine Clearance (ml/min)	999	85 ± 38	67 ± 32	**<0.001**
Diabetes Mellitus	999			**0.028**
No		642 (68.7%)	36 (55.4%)	
Diet-Controlled		44 (4.7%)	1 (1.5%)	
Tablet-Controlled		155 (16.6%)	19 (29.2%)	
Insulin-Dependent		93 (10.0%)	9 (13.8%)	
Hypertension	993	528 (56.9%)	48 (73.8%)	**0.009**
Hypercholesterolemia	917	412 (48.0%)	29 (49.2%)	0.893
Previous CVA	999	41 (4.4%)	8 (12.3%)	**0.011**
Previous Myocardial Infarction	996	198 (21.3%)	25 (38.5%)	**0.003**
Previous CABG	1000	77 (8.2%)	8 (12.3%)	0.250
Previous PCI	1000	164 (17.5%)	19 (29.2%)	**0.029**
Family History of CAD	966	377 (41.8%)	22 (34.4%)	0.293
Left Ventricular Ejection Fraction	928			0.008 *
Normal		645 (74.2%)	35 (59.3%)	
Mild Impairment		125 (14.4%)	10 (16.9%)	
Moderate Impairment		51 (5.9%)	9 (15.3%)	
Severe Impairment		48 (5.5%)	5 (8.5%)	
Presentation				
Symptom Onset to PCI (Hours)	1000	24 (5–81)	36 (5–107)	0.179
Out-of-Hospital Cardiac Arrest	1000	37 (4.0%)	4 (6.2%)	0.334
NSTEMI	1000	528 (56.5%)	41 (63.1%)	0.365
Index Troponin (ng/L)	941	91 (28–352)	94 (37–719)	0.275
Peak Troponin (ng/L)	987	512 (77–2512)	800 (169–3061)	0.155
Mitral Regurgitation Severity	1000			<0.001 *
None		683 (73.0%)	29 (44.6%)	
Mild		200 (21.4%)	22 (33.8%)	
Moderate		47 (5.0%)	10 (15.4%)	
Severe		5 (0.5%)	4 (6.2%)	
Pattern of CAD **	1000			0.322 *
No Severe Disease		8 (0.9%)	2 (3.1%)	
Single Vessel Disease		590 (63.1%)	35 (53.8%)	
Double Vessel Disease		242 (25.9%)	19 (29.2%)	
Triple Vessel Disease		95 (10.2%)	9 (13.8%)	
Left Main Stem Involvement ***	1000	64 (6.8%)	12 (18.5%)	**0.002**

Continuous variables are reported as either means ± standard deviations or medians (interquartile range) with *p*-values from Mann–Whitney U tests. Categorical variables are reported as N (%) with *p*-values from Fisher’s exact tests unless stated otherwise. **Bold** *p*-values are significant at *p* < 0.05. * *p*-value from Mann–Whitney U test, as the factor is ordinal. ** Defined as the number of vessels (from left anterior descending, left circumflex, and right coronary arteries) with ≥75% luminal stenosis. *** Defined as a ≥50 luminal stenosis. CABG: Coronary Artery Bypass Graft, CAD: Coronary Artery Disease, CVA: Cerebrovascular Accident, NSTEMI: Non-ST-Elevation Myocardial Infarction, and PCI: Percutaneous Coronary Intervention.

**Table 2:** Cohort characteristics organized by concomitant atrial fibrillation.

		Concomitant Atrial Fibrillation		
	N	No	Yes	*p*-Value
Angioplasty Details				
DTB for Primary PCI (Minutes) *	431	40 (28–75)	54 (34–79)	0.219
CABG	1000	31 (3.3%)	6 (9.2%)	**0.028**
Complication	1000	5 (0.5%)	1 (1.5%)	0.333
Failure	1000	20 (2.1%)	0 (0.0%)	0.635
Rotablation	1000	26 (2.8%)	4 (6.2%)	0.125
Circulatory Support	1000	3 (0.3%)	0 (0.0%)	1.000
Discharge Medication **				
Aspirin	948	870 (98.3%)	56 (88.9%)	**<0.001**
Clopidogrel	948	173 (19.5%)	32 (50.8%)	**<0.001**
Ticagrelor	948	649 (73.3%)	25 (39.7%)	**<0.001**
Prasugrel	948	48 (5.4%)	2 (3.2%)	0.767
NOAC	948	23 (2.6%)	21 (33.3%)	**<0.001**
Warfarin	948	14 (1.6%)	11 (17.5%)	**<0.001**
Beta Blocker	948	786 (88.8%)	53 (84.1%)	0.303
ACE Inhibitor	948	691 (78.1%)	38 (60.3%)	**0.003**
Angiotensin Receptor Blocker	948	93 (10.5%)	11 (17.5%)	0.095
Aldosterone Receptor Antagonist	948	75 (8.5%)	15 (23.8%)	**<0.001**
Diuretic	948	134 (15.1%)	30 (47.6%)	**<0.001**

Continuous variables are reported as either means ± standard deviations or medians (interquartile range) with *p*-values from Mann–Whitney U tests. Categorical variables are reported as N (%) with *p*-values from Fisher’s exact tests. **Bold** *p*-values are significant at *p* < 0.05. * Only includes patients with ST-elevation myocardial infarction. ** Excludes patients who died in hospital (N = 41) or for whom discharge letters were unavailable (N = 11). ACE: Angiotensin-Converting Enzyme, CABG: Coronary Artery Bypass Graft. DTB: Door-to-Balloon Time, NOAC: Non-Vitamin K Antagonist Oral Anticoagulants, and PCI: Percutaneous Coronary Intervention.

**Table 3:** Associations between AF and major adverse cardiac and cerebrovascular events.

	Number of Patients with Events		Kaplan–Meier Estimated Event Rate at Five Years		Hazard Ratio(95% CI)	
Outcome	Non-AF	AF	Non-AF	AF		*p*-Value
Any MACCE	329	35	34.2%	50.8%	1.86 (1.32–2.64)	<0.001
Death	228	30	23.3%	43.7%	2.25 (1.54–3.29)	<0.001
MACCE Readmission *	152	16	18.0%	31.0%	1.86 (1.11–3.11)	0.018
Myocardial Infarction Readmission **	93	10	10.8%	17.5%	1.86 (0.97–3.57)	0.063
Cerebrovascular Accident Readmission **	27	4	3.2%	7.8%	2.59 (0.91–7.41)	0.076
Major Gastrointestinal Bleed Readmission **	57	6	6.9%	11.8%	1.83 (0.79–4.24)	0.160

Numbers of patients with events represent the numbers of patients for whom the stated outcome occurred during follow-up. Hazard ratios and *p*-values are from univariable Cox regression models, and are reported for AF vs. non-AF patients. **Bold** *p*-values are significant at *p* < 0.05. * Readmission due to myocardial infarction, cerebrovascular accident, or major gastrointestinal bleed; follow-up was censored at death. ** Follow-up was censored at death. AF: Concomitant Atrial Fibrillation, 95% CI: 95% Confidence Interval, and MACCE: Major Adverse Cardiac and Cerebrovascular Event (defined as death or readmission due to myocardial infarction, cerebrovascular accident, or major gastrointestinal bleed).

**Table 4:** Associations with major adverse cardiac and cerebrovascular events.

	Univariable Models		Multivariable Model	
	HR (95% CI)	*p*-Value	HR (95% CI)	*p*-Value
Concomitant Atrial Fibrillation	1.86 (1.32–2.64)	**<0.001**	1.25 (0.81–1.92)	0.319
Age (Years) *		**<0.001**		**<0.001**
<50	-	-	-	-
50–59	1.24 (0.78–1.95)	0.361	0.88 (0.51–1.52)	0.646
60–69	1.48 (0.95–2.29)	0.081	0.83 (0.47–1.44)	0.497
70–79	2.19 (1.43–3.35)	**<0.001**	0.96 (0.53–1.72)	0.886
80+	4.36 (2.85–6.67)	**<0.001**	1.91 (1.04–3.49)	**0.037**
Gender (Female)	1.27 (1.02–1.58)	**0.033**	-	*NS*
Smoking Status		**0.002**		*NS*
Non-	-	-	-	-
Ex-	1.22 (0.96–1.55)	0.111	-	-
Current	0.74 (0.56–0.97)	**0.031**	-	-
Creatinine Clearance (per 10 mL/min)	0.86 (0.84–0.89)	**<0.001**	0.94 (0.90–0.99)	**0.011**
Diabetes Mellitus		**<0.001**		**<0.001**
No	-	-	-	-
Diet-Controlled	1.23 (0.75–2.01)	0.421	1.64 (0.95–2.84)	0.076
Tablet-Controlled	1.33 (1.01–1.76)	**0.040**	1.12 (0.79–1.57)	0.532
Insulin-Dependent	3.11 (2.37–4.09)	**<0.001**	2.42 (1.69–3.45)	**<0.001**
Hypertension	2.10 (1.67–2.64)	**<0.001**	1.72 (1.27–2.33)	**<0.001**
Hypercholesterolemia	1.00 (0.80–1.24)	0.979	-	*NS*
Previous CVA	2.70 (1.89–3.84)	**<0.001**	2.46 (1.61–3.76)	**<0.001**
Previous Myocardial Infarction	2.06 (1.65–2.56)	**<0.001**	1.50 (1.13–1.98)	**0.004**
Previous CABG	1.86 (1.37–2.52)	**<0.001**	-	*NS*
Previous PCI	1.48 (1.16–1.89)	**0.001**	-	*NS*
Family History of CAD	0.84 (0.68–1.05)	0.120	-	*NS*
Left Ventricular Ejection Fraction		**<0.001**		**0.009**
Normal	-	-	-	-
Mild Impairment	1.48 (1.10–2.00)	**0.011**	1.42 (1.00–2.01)	0.053
Moderate Impairment	2.38 (1.65–3.44)	**<0.001**	1.70 (1.09–2.63)	**0.019**
Severe Impairment	2.98 (2.08–4.29)	**<0.001**	1.80 (1.13–2.86)	**0.013**
Symptom Onset to PPCI (per Day)	1.01 (1.00–1.03)	0.072	-	*NS*
Out-of-Hospital Cardiac Arrest	1.42 (0.90–2.26)	0.135	-	*NS*
NSTEMI	1.16 (0.94–1.43)	0.166	-	*NS*
Index Troponin (per 1000 ng/L)	1.03 (0.95–1.12)	0.479	-	*NS*
Peak Troponin (per 1000 ng/L)	1.00 (0.96–1.04)	0.920	-	*NS*
Mitral Regurgitation Severity		**<0.001**		**0.042**
None	-	-	-	-
Mild	1.23 (0.96–1.57)	0.100	0.78 (0.57–1.06)	0.112
Moderate/Severe **	2.14 (1.53–3.00)	**<0.001**	1.35 (0.88–2.09)	0.174
Pattern of CAD		**<0.001**		**0.007**
None/Single Vessel **	-	-	-	-
Double Vessel	1.21 (0.95–1.54)	0.122	0.85 (0.63–1.15)	0.285
Triple Vessel	2.52 (1.90–3.35)	**<0.001**	1.60 (1.12–2.28)	**0.010**
Left Main Stem Involvement	1.70 (1.22–2.38)	**0.002**	-	*NS*

Initially, individual univariable Cox regression models were produced for each factor. All factors were then considered alongside concomitant atrial fibrillation for inclusion in a multivariable Cox regression model, with a backward stepwise approach being used to produce a parsimonious model. The final model was based on N = 772 cases (N = 263 events) after exclusion of patients with missing data for any of the factors considered. Hazard ratios for categorical variables are reported for the stated category relative to the reference category. For continuous variables, hazard ratios are reported per increase in the stated number of units. **Bold** *p*-values are significant at *p* < 0.05. * Age was divided into categories for analysis as goodness-of-fit testing indicated poor model fit when it was treated as continuous. ** Categories were combined for analysis due to small within-category sample sizes. CABG: Coronary Artery Bypass Graft, 95% CI: 95% Confidence Interval, CAD: Coronary Artery Disease, CVA: Cerebrovascular Accident, HR: Hazard Ratio, NS: Not Selected by the Stepwise Procedure, NSTEMI: Non-ST-Elevation Myocardial Infarction, and (P)PCI: (Primary) Percutaneous Coronary Intervention.

## Data Availability

The data presented in this study are available on request from the corresponding author.

## Supplementary Materials

The following supporting information can be downloaded at: https://www.mdpi.com/article/10.3390/jcm13082318/s1, Table S1: ICD 10 codes used to identify adverse outcomes. Table S2: Characteristics of the propensity matched cohort.

## References

[1] Lip G.Y.H., Tse H.-F. (2007). Management of Atrial Fibrillation. Lancet.

[2] Miyasaka Y., Barnes M.E., Gersh B.J., Cha S.S., Bailey K.R., Abhayaratna W.P., Seward J.B., Tsang T.S.M. (2006). Secular trends in incidence of atrial fibrillation in Olmsted County, Minnesota, 1980 to 2000, and implications on the projections for future prevalence. Circulation.

[3] Schnabel R.B., Yin X., Gona P., Larson M.G., Beiser A.S., McManus D.D., Newton-Cheh C., A Lubitz S., Magnani J.W., Ellinor P.T. (2015). 50 year trends in atrial fibrillation prevalence, incidence, risk factors, and mortality in the Framingham Heart Study: A cohort study. Lancet.

[4] Go A.S., Hylek E.M., Phillips K.A., Chang Y., Henault L.E., Selby J.V., Singer D.E. (2001). Prevalence of Diagnosed Atrial Fibrillation in Adults. JAMA.

[5] Michniewicz E., Mlodawska E., Lopatowska P., Tomaszuk-Kazberuk A., Malyszko J. (2018). Patients with atrial fibrillation and coronary artery disease—Double trouble. Adv. Med. Sci..

[6] Violi F., Soliman E.Z., Pignatelli P., Pastori D. (2016). Atrial fibrillation and myocardial infarction: A systematic review and appraisal of pathophysiologic mechanisms. J. Am. Heart Assoc..

[7] Schmitt J., Duray G., Gersh B.J., Hohnloser S.H. (2008). Atrial fibrillation in acute myocardial infarction: A systematic review of the incidence, clinical features and prognostic implications. Eur. Heart J..

[8] Soliman E.Z., Safford M.M., Muntner P., Khodneva Y., Dawood F.Z., Zakai N.A., Thacker E.L., Judd S., Howard V.J., Howard G. (2014). Atrial Fibrillation and the Risk of Myocardial Infarction. JAMA Intern. Med..

[9] Bayturan O., Puri R., Tuzcu E.M., Shao M., Wolski K., Schoenhagen P., Kapadia S., E Nissen S., Sanders P., Nicholls S.J. (2016). Atrial fibrillation, progression of coronary atherosclerosis and myocardial infarction. Eur. J. Prev. Cardiol..

[10] Lee H.Y., Yang P.-S., Kim T.-H., Uhm J.-S., Pak H.-N., Lee M.-H., Joung B. (2017). Atrial fibrillation and the risk of myocardial infarction: A nation-wide propensity-matched study. Sci. Rep..

[11] Wong C.-K., White H.D., Wilcox R.G., Criger D.A., Califf R.M., Topol E.J., Ohman E. (2000). New atrial fibrillation after acute myocardial infarction independently predicts death: The GUSTO-III experience. Am. Heart J..

[12] Jabre P., Roger V.L., Murad M.H., Chamberlain A.M., Prokop L., Adnet F., Jouven X. (2011). Mortality associated with atrial fibrillation in patients with myocardial infarction. Circulation.

[13] Konttila K.K., Punkka O., Koivula K., Eskola M.J., Martiskainen M., Huhtala H., Virtanen V.K., Mikkelsson J., Järvelä K., Laurikka J. (2021). The Effect of Atrial Fibrillation on the Long-Term Mortality of Patients with Acute Coronary Syndrome: The TACOS Study. Cardiology.

[14] Madsen J.M., Jacobsen M.R., Sabbah M., Topal D.G., Jabbari R., Glinge C., Køber L., Torp-Pedersen C., Pedersen F., Sørensen R. (2021). Long-term prognostic outcomes and implication of oral anticoagulants in patients with new-onset atrial fibrillation following st-segment elevation myocardial infarction. Am. Heart J..

[15] Køber L., Swedberg K., McMurray J.J., Pfeffer M.A., Velazquez E.J., Diaz R., Maggioni A.P., Mareev V., Opolski G., Van de Werf F. (2006). Previously known and newly diagnosed atrial fibrillation: A major risk indicator after a myocardial infarction complicated by heart failure or left ventricular dysfunction. Eur. J. Heart Fail..

[16] Crenshaw B.S., Ward S.R., Granger C.B., Stebbins A.L., Topol E.J., Califf R.M. (1997). Atrial fibrillation in the setting of acute myocardial infarction. J. Am. Coll. Cardiol..

[17] Eldar M., Canetti M., Rotstein Z., Boyko V., Gottlieb S., Kaplinsky E., Behar S. (1998). Significance of Paroxysmal Atrial Fibrillation Complicating Acute Myocardial Infarction in the Thrombolytic Era. Circulation.

[18] Hindricks G., Potpara T., Dagres N., Arbelo E., Bax J.J., Blomstrom-Lundqvist C., Boriani G., Castella M., Dan G.A., Dilaveris P.E. (2021). Corrigendum to: 2020 ESC guidelines for the diagnosis and management of atrial fibrillation developed in collaboration with the European Association for Cardio-Thoracic Surgery (EACTS): The Task Force for the diagnosis and management of atrial fibrillation of the European Society of Cardiology (ESC) developed with the special contribution of the European Heart Rhythm Association (EHRA) of the ESC. Eur. Heart J..

[19] Kinjo K., Sato H., Sato H., Ohnishi Y., Hishida E., Nakatani D., Mizuno H., Fukunami M., Koretsune Y., Takeda H. (2003). Prognostic significance of atrial fibrillation/atrial flutter in patients with acute myocardial infarction treated with percutaneous coronary intervention. Am. J. Cardiol..

[20] Motloch L.J., Reda S., Larbig R., Wolff A., Motloch K.A., Wernly B., Granitz C., Lichtenauer M., Wolny M., Hoppe U.C. (2017). Characteristics of coronary artery disease among patients with atrial fibrillation compared to patients with sinus rhythm. Hell. J. Cardiol..

[21] Alasady M., Abhayaratna W.P., Leong D.P., Lim H.S., Abed H.S., Brooks A.G., Mattchoss S., Roberts-Thomson K.C., Worthley M.I., Chew D.P. (2011). Coronary artery disease affecting the atrial branches is an independent determinant of atrial fibrillation after myocardial infarction. Heart Rhythm.

[22] Obayashi Y., Shiomi H., Morimoto T., Tamaki Y., Inoko M., Yamamoto K., Takeji Y., Tada T., Nagao K., Yamaji K. (2021). Newly Diagnosed Atrial Fibrillation in Acute Myocardial Infarction. J. Am. Heart Assoc..

