# Supplementary material for: Prevalence and Impact of Concomitant Atrial Fibrillation in Patients Undergoing Percutaneous Coronary Intervention for Acute Myocardial Infarction
Source: J Clin Med. 2024 Apr 17;13(8):2318. doi: 10.3390/jcm13082318 (PMC11050934; doi:10.3390/jcm13082318)
Supplement: Supplementary file 1 [file jcm-13-02318-s001.zip › jcm-2907463-supplementary.pdf]

***Supplementary Table S1 ICD 10 codes used to identify adverse outcomes***

**Myocardial Infarction**
- I21 - Acute myocardial infarction
- I22 - Subsequent myocardial infarction

**Cerebrovascular Accident**
- I60 - Subarachnoid haemorrhage
- I61 - Intracerebral haemorrhage
- I63 - Cerebral infarction
- I64 - Stroke, not specified as haemorrhage or infarction

**Major Gastrointestinal Bleeding**
- K920 - Haematemesis
- K921 - Melaena
- K922 - Gastrointestinal haemorrhage, unspecified
- K226 - Gastro-oesophageal laceration-haemorrhage syndrome
- K228 - Other specified diseases of oesophagus
- K2(5,6,7,8)0 - (Gastric, Duodenal, Peptic, Gastrojejunal ulcer) acute with haemorrhage
- K2(5,6,7,8)2 - (Gastric, Duodenal, Peptic, Gastrojejunal ulcer) acute with both haemorrhage and perforation
- K2(5,6,7,8)4 - (Gastric, Duodenal, Peptic, Gastrojejunal ulcer) chronic or unspecified with haemorrhage
- K2(5,6,7,8)6 - (Gastric, Duodenal, Peptic, Gastrojejunal ulcer) chronic or unspecified with both haemorrhage and perforation
- K290 - Acute haemorrhagic gastritis
- K552 - Angiodysplasia of colon
- K625 - Haemorrhage of anus and rectum
- I850 - Oesophageal varices with bleeding
- I983 - Oesophageal varices with bleeding in diseases classified elsewhere

***Supplementary Table S2 Characteristics of the propensity matched cohort***

| | N Pairs | Concomitant Atrial Fibrillation *No* | *Yes* | p-Value |
|---|---|---|---|---|
| **Patient Demographics** | | | | |
| Age (Years) | 65 | 71 ± 11 | 73 ± 11 | 0.191 |
| Gender (% Male) | 65 | 50 (77%) | 49 (75%) | 1.000 |
| Smoking Status** | 55 | | | 0.326 |
| *Non-* | | 22 (40%) | 22 (40%) | |
| *Ex-* | | 19 (35%) | 22 (40%) | |
| *Current* | | 14 (25%) | 11 (20%) | |
| Creatinine Clearance (ml/min) | 65 | 66 ± 30 | 67 ± 32 | 0.966 |
| Diabetes Mellitus | 65 | | | 0.543 |
| *No* | | 41 (63%) | 36 (55%) | |
| *Diet-Controlled* | | 1 (2%) | 1 (2%) | |
| *Tablet-Controlled* | | 16 (25%) | 19 (29%) | |
| *Insulin-Dependent* | | 7 (11%) | 9 (14%) | |
| Hypertension | 65 | 51 (78%) | 48 (74%) | 0.678 |
| Hypercholesterolemia** | 55 | 34 (62%) | 27 (49%) | 0.248 |
| Previous CVA | 65 | 11 (17%) | 8 (12%) | 0.607 |
| Previous Myocardial Infarction | 65 | 19 (29%) | 25 (38%) | 0.327 |
| Previous CABG | 65 | 11 (17%) | 8 (12%) | 0.607 |
| Previous PCI | 65 | 15 (23%) | 19 (29%) | 0.481 |
| Family History of CAD | 64 | 26 (41%) | 22 (34%) | 0.572 |
| Left Ventricular Ejection Fraction** | 54 | | | 0.447* |
| *Normal* | | 36 (67%) | 30 (56%) | |
| *Mild Impairment* | | 6 (11%) | 10 (19%) | |
| *Moderate Impairment* | | 6 (11%) | 9 (17%) | |
| *Severe Impairment* | | 6 (11%) | 5 (9%) | |

| Presentation | | | | |
|---|---|---|---|---|
| Symptom Onset to PPCI (Hours) | 65 | 46 (7-123) | 36 (5-107) | 0.433 |
| Out-of-Hospital Cardiac Arrest | 65 | 5 (8%) | 4 (6%) | 1.000 |
| NSTEMI | 65 | 45 (69%) | 41 (63%) | 0.597 |
| Index Troponin (ng/L)*** | 55 | 165 (43-477) | 121 (38-841) | 0.960 |
| Peak Troponin (ng/L) | 64 | 616 (167-3055) | 800 (169-3061) | 0.748 |
| Mitral Regurgitation Severity | 65 | | | 0.435* |
| *None* | | 24 (37%) | 29 (45%) | |
| *Mild* | | 27 (42%) | 22 (34%) | |
| *Moderate/Severe* | | 14 (22%) | 14 (22%) | |
| Pattern of CAD | 65 | | | 0.181* |
| *None/Single Vessel Disease* | | 28 (43%) | 37 (57%) | |
| *Double Vessel Disease* | | 25 (38%) | 19 (29%) | |
| *Triple Vessel Disease* | | 12 (18%) | 9 (14%) | |
| Left Main Stem Involvement | 65 | 14 (22%) | 12 (18%) | 0.804 |

*Results are for the N=65 pairs of propensity-score-matched patients unless stated otherwise. In cases of missing data, only those pairs where data were available for both patients were included in the analysis. Continuous variables are reported as either means ± standard deviations or medians (interquartile range) with p-values from Wilcoxon's signed rank tests. Categorical variables are reported as N (%) with p-values from McNemar's test/McNemar–Bowker tests unless stated otherwise. Bold p-values are significant at p<0.05. *p-value from Wilcoxon's signed-rank test, as the factor is ordinal. **Data were missing for >1 patient in the AF group; these patients were combined into a "missing data" group when producing the propensity score to prevent exclusions of patients. ***Data were missing for >1 patient in the AF group; since index troponin was strongly correlated with peak troponin, it was not considered for inclusion in the propensity score model to prevent exclusions of AF patients. CABG: Coronary Artery Bypass Graft, CAD: Coronary Artery Disease, CVA: Cerebrovascular Accident, NSTEMI: Non-ST-Elevation Myocardial Infarction, and (P)PCI: (Primary) Percutaneous Coronary Intervention.*
